# Late-Onset Epilepsy With Unknown Etiology: A Pilot Study on Neuropsychological Profile, Cerebrospinal Fluid Biomarkers, and Quantitative EEG Characteristics

**DOI:** 10.3389/fneur.2020.00199

**Published:** 2020-04-15

**Authors:** Elena Nardi Cesarini, Claudio Babiloni, Nicola Salvadori, Lucia Farotti, Claudio Del Percio, Maria Teresa Pascarelli, Giuseppe Noce, Roberta Lizio, Fulvio Da Re, Valeria Isella, Lucio Tremolizzo, Michele Romoli, Jacopo C. DiFrancesco, Lucilla Parnetti, Cinzia Costa

**Affiliations:** ^1^Neurology Clinic, University of Perugia–S. Maria della Misericordia Hospital, Perugia, Italy; ^2^Department of Physiology and Pharmacology “V. Erspamer,” Faculty of Pharmacy and Medicine, Sapienza University of Rome, Rome, Italy; ^3^Hospital San Raffaele Cassino, Cassino, Italy; ^4^Oasi Research Institute - IRCCS, Troina, Italy; ^5^IRCCS SDN, Naples, Italy; ^6^Department of Neurology, Milan Center for Neuroscience, School of Medicine and Surgery, San Gerardo Hospital, University of Milano-Bicocca, Monza, Italy; ^7^Neurology Unit, Rimini “Infermi” Hospital–AUSL Romagna, Rimini, Italy

**Keywords:** late onset epilepsy of unknown etiology, mild cognitive impairment, neuropsychology, CSF biomarkers, quantitative EEG

## Abstract

**Introduction:** Despite the fact that epilepsy has been associated with cognitive decline, neuropsychological, neurobiological, and neurophysiological features in patients with late-onset epilepsy of unknown etiology (LOEU) are still unknown. This cross-sectional study aims to investigate the neuropsychological profile, cerebrospinal fluid (CSF) biomarkers of Alzheimer's disease (AD), and resting-state quantitative electroencephalographic (qEEG) cortical rhythms in LOEU patients with mild cognitive impairment (LOEU-MCI) and with normal cognition (LOEU-CN), compared to non-epileptic MCI (NE-MCI) and cognitively normal (CN) controls.

**Methods:** Consecutive patients in two clinical Units diagnosed with LOEU-CN (19), LOEU-MCI (27), and NE-MCI (21) were enrolled, and compared to age and sex-matched cognitively normal subjects CN (11). Patients underwent standardized comprehensive neuropsychological evaluation and CSF core AD biomarkers assessment (i.e., CSF Aβ42, phospho-tau and total tau, classified through A/T/(N) system). Recordings of resting-state eyes-closed electroencephalographic (EEG) rhythms were collected and cortical source estimation of delta (<4 Hz) to gamma (>30 Hz) bands with exact Low Resolution Electromagnetic Tomography (eLORETA) was performed.

**Results:** Most LOEU patients had an MCI status at seizure onset (59%). Patients with LOEU-MCI performed significantly worse on measures of global cognition, visuo-spatial abilities, and executive functions compared to NE-MCI patients (*p* < 0.05). Regarding MCI subtypes, multiple-domain MCI was 3-fold more frequent in LOEU-MCI than in NE-MCI patients (OR 3.14, 95%CI 0.93–10.58, *p* = 0.06). CSF Aβ42 levels were lower in the LOEU-MCI compared with the LOEU-CN group. Finally, parietal and occipital sources of alpha (8–12 Hz) rhythms were less active in the LOEU-MCI than in the NE-MCI and CN groups, while the opposite was true for frontal and temporal cortical delta sources.

**Discussion:** MCI status was relatively frequent in LOEU patients, involved multiple cognitive domains, and might have been driven by amyloidosis according to CSF biomarkers. LOEU-MCI status was associated with abnormalities in cortical sources of EEG rhythms related to quiet vigilance. Future longitudinal studies should cross-validate our findings and test the predictive value of CSF and EEG variables.

## Introduction

Epilepsy affects 65 million people worldwide (1), with increasing prevalence after age 55 (2, 3). The population of older adults with epilepsy consists of two main groups: those who have had epilepsy for many years and those who develop epilepsy *de novo* in later life (4), also known as late-onset epilepsy (LOE).

Several causes may underlie LOE, the most common being cerebrovascular disease (up to 50% of cases), head injury (20% of cases), and brain tumors (5). However, patients with Alzheimer's disease (AD) are up to 10 times more likely to develop LOE than those without AD (4). Furthermore, dementia due to AD and other etiologies are estimated to account for 10–20% of LOE (5). However, despite the bulk of the literature focused on dementia as startling cause of epilepsy in elderly (6–9) as well as on cognitive performance in young onset epilepsy (10–13), little is known on cognition among people with LOE (4). Indeed, only isolated reports are available, yet they do not providing the prevalence and characterization of cognitive impairment in people who have received a LOE diagnosis (4, 14–16). Such issues, though under-investigated, might offer critical insights to define the processes shared by epileptogenesis and neurodegeneration. An intertwining that becomes critical for patients with late onset epilepsy of unknown etiology (LOEU), that make up 20% of LOE, and among which extensive investigations yield no vascular, structural, or systemic etiology (15, 17).

Despite the fact that the role of beta-amyloid (β-amyloid) has recently been postulated (14, 15, 17), we are still far from grasping the whole clinical, cognitive, neurobiological, and neurophysiological profile in patients with LOEU.

The present cross-sectional study aimed to investigate the neuropsychological profile, CSF biomarkers of Aβ42, total tau [t-tau] and phosphorylated tau [p-tau], and resting-state quantitative EEG (qEEG) cortical rhythms in LOEU patients, comparing them to non-epileptic controls, including MCI (NE-MCI) and cognitively normal (CN) subjects.

## Materials and Methods

### Cohorts

A consecutive series of patients aged >55 years diagnosed with LOE at the Neurology of the University of Perugia and at the San Gerardo Hospital of Monza (Italy) between 2018 and 2019 was included. The protocol was approved by the Ethical Board (WP5 P001; N 2049/12) and informed consent was obtained for the study procedure (15, 17). Epilepsy diagnosis, seizure type, and EEG patterns were characterized according to the International League Against Epilepsy (ILAE) Classification criteria (18). At baseline, patients underwent medical history examination, clinical examination by experienced neurologists, blood chemistry testing, EEG and brain MRI, and extensive standardized neuropsychological assessment, according to a previously defined protocol, through which alternative causes of epilepsy were ruled out, leading to the diagnosis of LOEU (15). Inclusion criteria were: (i) LOEU diagnosis, (ii) no previous or current medical history of other significant neurological or psychiatric disorders, (iii) no previous or current use of acetylcholinesterase inhibitors or antipsychotic drugs/lithium, (iv) non-demented status [Clinical Dementia Rating (CDR) scale <1]. According to cognitive testing (see below), LOEU patients were further grouped into LOEU with MCI (LOEU-MCI) and LOEU with normal cognition (LOEU-CN). After obtaining written consent, patients underwent a lumbar puncture (LP) for CSF core AD biomarkers analysis (Aβ42, t-tau and p-tau).

Age and sex-matched non-epileptic MCI (NE-MCI) patients followed the same extensive work-up designed for LOEU patients, allowing for a direct comparison of neuropsychological testing scores, CSF biomarkers, and EEG findings.

Finally, a control group of age- and sex-matched non-epileptic cognitively normal (CN) subjects was drawn from a consecutive series of patients undergoing extensive diagnostics for other neurological conditions. All patients in this group received the abovementioned diagnostics.

The main aims were: (i) defining cognitive status at LOEU diagnosis, (ii) comparing cognitive performance depending on epilepsy status and MCI status, (iii) evaluating differences in CSF biomarkers in LOEU vs. NE-MCI and CN subjects, as well as in LOEU-MCI vs. LOEU-CN, and (iv) identifying rsEEG abnormalities in LOEU.

### Neuropsychological Evaluation

A neuropsychological evaluation assessing global cognition, memory, attention/executive functions, language, and visuospatial skills was performed within a month of LOEU diagnosis. Specifically, global cognition was assessed using the Mini-Mental State Examination (MMSE) (19). For the assessment of the memory domain, we administered the Rey Auditory Verbal Learning Test (RAVLT) (20). The tests assessing attention and executive functions included the Trail Making Test (TMT) part A and B (21) and the Frontal Assessment Battery (FAB) (22). The tests assessing language included the 1-min verbal fluency both for letters (FAS) (20) and semantic categories (fruits, animals, and car trades) (23). Abstract logical reasoning was assessed by Raven's Colored Progressive Matrices'47 (PM'47) (24). Finally, the tests assessing visuo-spatial skills included the copy of drawings with and without landmarks from the Mental Deterioration Battery (MDB) (20) and the Clock Drawing Test (CDT) (25). The Clinical Dementia Rating (CDR) scale (26) was used to stage clinical status. MCI was diagnosed and classified according to clinical and neuropsychological criteria (27) as follows: (i) if only the memory domain was impaired the patient was designated as having amnestic-single-domain MCI (a-sd MCI), (ii) if other domains were involved beyond memory, the patient was diagnosed with amnestic-multi-domain MCI (a-md MCI); (iii) if cognitive impairment involved one or more domains other than memory, a diagnosis of non-amnestic MCI (na-MCI) was postulated, including both the non-amnestic single domain MCI (na-sd-MCI) and non-amnestic multi-domain MCI (na-md-MCI).

### CSF Biomarkers and Diagnostics

CSF samples were collected in the morning in fasting patients by lumbar puncture between the L3/L4 or L4/L5 intervertebral space and were analyzed at the University Hospital of Perugia Lab of Clinical Neurochemistry and at the dedicated Lab at the San Gerardo Hospital. Lumbar puncture was performed within 2 months after epilepsy diagnosis, and at least 3 weeks after the last seizure, in all patients accepting to undergo the procedure (LOEU group *n* = 30, 63%). All NE-MCI and CN underwent lumbar puncture. CSF analysis included routine chemical physical parameters (glucose, total proteins, albumin, and cell count) and the measurement of classical AD biomarkers (Aβ42, t-tau, p-tau) by means of enzyme-linked immunosorbent assay kits (Fujirebio), according to a standardized protocol (28). The AT(N) scoring system was applied to identify changes in β-amyloid pathology (A) and tauopathy (T), with Alzheimer's pathological changes defined as β-amyloid pathology [A+] independently from tauopathy, and AD defined as A+ T+ (29).

### Video EEG Recordings and Quantitative EEG Analysis

EEG recordings took place during routine 20-min sessions within 1 month after epilepsy diagnosis. The subjects were seated in a reclined bed in a fully lit room, with sound attenuation. They were relaxed with eyes closed (no photic and hyper-ventilatory stimulation). Video-EEG recordings were performed using 21-electrodes of the standard international 10–20 electrode placement (30). EEG technicians and physicians supervising the EEG recordings monitored it carefully, alerting the patients by sound stimuli at first signs of drowsiness. They were blinded for epilepsy diagnosis, CSF biomarkers, neuropsychological assessment results, and cognitive status.

Quantitative EEG analysis was performed at the University of Rome “Sapienza” (31), to analyze cortical connectivity and neuronal synchronization of rhythmic oscillations at various frequencies. Briefly, the resting state EEG (rsEEG) data were segmented and analyzed offline in consecutive 2 s epochs. Artifactual epochs were identified using a computerized home-made automatic software procedure (32), confirmed by two EEG experts and then eliminated. Artifact-free rsEEG epochs recorded during the eyes-open condition were used to control the expected reactivity of alpha rhythms as a sign of good quality of rsEEG recordings. Artifact-free rsEEG epochs recorded during the eyes-closed condition were used as an input for the analysis of the EEG power density spectrum.

Due to the suitability of recordings at post-processing, qEEG was limited to the comparison of LOEU-MCI and NE-MCI patients with CSF Aβ42/p-tau levels lower than 15.2 for APOE ε 4 carriers, and 8.9 for APOE ε 4 non-carriers (33) and CN subjects. In particular, official exact Low-Resolution Brain Electromagnetic Tomography (eLORETA) (34) freeware was used for the estimation of cortical sources of the rsEEG. eLORETA estimated the activity of global and regional (i.e., frontal, central, parietal, occipital, temporal, and limbic lobes as defined in the eLORETA brain atlas) normalized cortical (eLORETA) sources of rsEEG rhythms for delta (2–4 Hz), theta (4–7 Hz), alpha 1 (8–10.5 Hz), alpha 2 (10.5–12), alpha 3 (12–13 Hz), beta 1 (13–20 Hz), beta 2 (20–30 Hz) and gamma (30–40 Hz) bands, as indexes of cortical neural synchronization (31). Mean values of the eLORETA cortical source activity of resting state eyes-closed EEG rhythms for band and region of interest (ROI) (central, frontal, parietal, occipital, temporal, limbic) were compared between CN patients and LOEU-MCI patients, and NE-MCI patients with a decreased Aβ42/p-tau ratio, according to a previously reported paradigm (31, 33).

We used the official freeware tool, called exact LORETA (eLORETA), for the inverse estimation of cortical source activities generating scalp-recorded rsEEG rhythms (34). The present implementation of eLORETA uses a spherical head volume conductor model composed of the scalp, skull, and brain compartments. In the scalp compartment, exploring electrodes can be virtually positioned to give EEG data as an input to the inverse source estimation (34). The cortical source space is formed by 6,239 voxels with a 5 mm resolution, restricted to the gray matter of the head volume conductor model. An equivalent current dipole is located in each voxel. In the eLORETA freeware (34), the cortical source model is co-registered with a realistic cerebral shape taken from a template typically used in neuroimaging studies, namely that of the Montreal Neurological Institute (MNI152 template). The eLORETA solutions are computed frequency bin-by-frequency bin at any voxel of the cortical source model from the EEG spectral power density computed at 19 scalp electrodes. For each voxel, the eLORETA package provides the Talairach coordinates, the lobe, and the Brodmann area (BA).

In line with the general low spatial resolution of the present EEG methodological approach (i.e., 19 scalp electrodes), we performed a regional rather than voxel-based analysis of the eLORETA solutions. For this purpose, we collapsed the eLORETA solutions at the voxels of the frontal, central, temporal, parietal, occipital, and limbic macro regions considered separately (31).

### Statistical Analysis

Statistical analysis was performed using SPSS v.25. Besides the first comparative study with multimodal testing, no previous study was available for accurate power calculation. From our preliminary data (15), we assumed that a sample size of 44 patients with LOEU and 20 non-epileptic patients would be needed to detect a 30% significant (*p* < 0.05) difference in amyloid pathology on CSF (power 80%). Continuous variables were described by means and standard deviations, while categorical ones were summarized with counts and percentages. Differences in continuous variables were tested with Student's *t*-test or Mann–Whitney *U*-test wherever appropriate, while differences of categorical variables were tested with the chi-square test or Fisher's exact test when appropriate (*p* < 0.05). For multiple comparisons of neuropsychological test scores among groups, Bonferroni correction was applied (*p* < 0.05). Cohen's d was calculated to estimate effect magnitude for neuropsychological tests reaching statistical difference between groups and reported in tables as low (<0.25), mild (0.25–0.74), moderate (0.75–0.99) and high (≥1). A comparison of continuous EEG variables in the LEOU-MCI group compared with the CN and NE-MCI groups was performed with the analysis of variance (ANOVA) and *post-hoc* Tukey's test (*p* < 0.05).

## Results

### Demographics

Overall, 78 subjects (46 LOEU, 21 NE-MCI, and 11 CN) were enrolled. Age, gender distribution, and education did not differ across groups (Table 1). In the LOEU group (*n* = 46, 43.5% female), seizure semiology was mostly focal (*n* = 40, 87%), and patients mostly received monotherapy (87%), with levetiracetam being the most commonly prescribed antiseizure medication (*n* = 27, 34.6%).

**Table 1:** Characteristics of cohorts.

		**Group**			**Overall**
		**LOEU**	**NE-MCI**	**CN**	
*n*		46	21	11	78
Gender (female), *n* (%)		20 (43.5%)	12 (57.1%)	4 (36.4%)	36 (46.2%)
Age, mean ±SD		67.5 ± 6.8	68.4 ± 7.4	65.7 ± 7.7	67.7 ± 7
Education, mean ± SD (years)		9.5 ± 4.4	10.3 ± 4.4	9.8 ± 4.0	9.7 ± 4.3
CSF biomarkers, mean ± SD					
	Aβ42 (pg/mL)	1028 ± 495.2	820 ± 316.6	1022.4 ± 196.3	956.6 ± 406.8
	t-tau (pg/mL)	326.4 ± 181.2	440.1 ± 238.7	298.5 ± 135	360 ± 201.8
	p-tau (pg/mL)	53.4 ± 26.1	70.4 ± 31.8	49.7 ± 20.1	58.5 ± 28.2
	Aβ42/p-tau ratio	24.3 ± 14.7	14.4 ± 8.8^**a**^	24.9 ± 13.1	21.1 ± 13.4

LOEU, late onset epilepsy of unknown etiology; CN, cognitive normal; NE-MCI, non epileptic-mild cognitive impairment; SD, standard deviation; CSF, cerebrospinal fluid.

a: p < 0.05 comparing with LOEU.

*One-way ANOVA, Tukey's post-hoc test, Bonferroni correction applied*.

In the LOEU group, 19 patients were classified as LOEU-CN and 27 patients as LOEU-MCI (Table 2). Prevalence of MCI patients in the LOEU group was 58.7%. LOEU-MCI patients were older at seizure onset (69.2 vs. 65.1 years) and had lower education (8.3 vs. 11.1) compared to LOEU-CN patients (*p* < 0.05). EEG, clinical history and seizure semiology were similar across groups. No differences in the standard assessment of EEG findings, including epileptic abnormalities and focal slowing, were reported.

**Table 2:** Late onset epilepsy of unknown etiology cohort characteristics.

		**LOEU patients**				
		**LOEU-CN**	**LOEU-MCI**	**Overall**
*n*		19	27	46
Age at seizure onset, mean ± SD (years)		65.1 ± 6.8	69.2 ± 6.4^*^	67.5 ± 6.8
Gender (female), *n* (%)		7 (36.8%)	13 (48.1%)	20 (43.5%)
Education, mean ± SD (years)		11.1 ± 4	8.3 ± 4.4^*^	9.5 ± 4.4
Seizure semiology	Focal	18 (94.7%)	22 (81.5%)	40 (87%)
	Generalized	1 (5.3%)	5 (18.5%)	6 (13%)
EEG				
	Epileptic abnormalities	5 (26.3%)	11 (40.7%)	16 (34.8%)
	Slowing	4 (21.1%)	4 (14.8%)	8 (17.4%)
CSF biomarkers, mean ±SD				
	Aβ42 (pg/mL)	1387.8 ± 671.6	897.2 ± 347.9^*^	1028 ± 495.2
	t-tau (pg/mL)	247.4 ± 92.1	355.1 ± 198.2	326.4 ± 181.2
	p-tau (pg/mL)	43.1 ± 11.3	57.1 ± 29.1	53.4 ± 26.1
	Aβ42/p-tau ratio	31 ± 8.7	21.9 ± 15.8	24.3 ± 14.7
Neuropsychological assessment scores, mean ±SD				
	MMSE	28.3 ± 1.5	25.6 ± 2.2^*^^#^	26.7 ± 2.3
	CDT	1.2 ± 1.6	4.6 ± 3.1^*^^#^	2.8 ± 3
	DIGIT F	6.3 ± 1.3	5.1 ± 1.1^*^^#^	5.6 ± 1.3
	DIGIT B	4.6 ± 0.9	2.8 ± 1.5^*^^#^	3.6 ± 1.5
	RAVLT imm	39.7 ± 8	28.8 ± 7.2^*^^#^	33.4 ± 9.2
	RAVLT del	7.9 ± 2.6	4 ± 3.3^*^^#^	5.6 ± 3.5
	RAVLT TR	13.4 ± 1.5	11.9 ± 2.7^*^^##^	12.5 ± 2.4
	RAVLT FP	1.4 ± 2.1	4.7 ± 4.2^*^^#^	3.4 ± 3.9
	TMT A	38.4 ± 11.5	83.8 ± 45^*^^#^	61.1 ± 39.7
	TMT B	123 ± 46.4	218.8 ± 77.7^*^^#^	165.9 ± 78
	TMT B-A	90.9 ± 32.4	115.3 ± 53.5	100.3 ± 42.5
	FAB	16.6 ± 1.4	13.3 ± 2.3^*^^#^	15 ± 2.5
	FAS	35.6 ± 9.4	19.8 ± 7^*^^#^	26.4 ± 11.2
	CF	36.1 ± 7.6	28.3 ± 7.8^*^^#^	32.2 ± 8.6
	PM'47	29.8 ± 3.6	23.5 ± 4.6^*^^#^	26.1 ± 5.2
	CD	9.8 ± 1.7	8.1 ± 2^*^^#^	8.8 ± 2.1
	CD L	67.8 ± 3.8	58.2 ± 12.8^*^^#^	61.8 ± 11.3

LOEU, late onset epilepsy of unknown etiology; CN, cognitively normal; MCI, mild cognitive impairment; SD, standard deviation; EEG, electroencephalogram; CSF, cerebrospinal fluid; MMSE, Mini Mental State Examination; CDT, Clock Drawing Test; Digit F, Digit Span Forward; Digit B, Digit Span Backward; RAVLT, Rey Auditory Verbal Learning Test (Imm, Immediate Recall; Del, Delayed Recall; TR, True Recognition; FP, False Positives). TMT, Trail Making Test; FAB, Frontal Assessment Battery; FAS, Phonemic/Letter Fluency (FAS); CF, Category Fluency; PM'47, Raven Colored Progressive Matrices'47; CD, Copying Drawings; CD L, Copying drawings with landmarks.

* p < 0.05.

# Cohen's d > 0.75 (range 0.8–1.4),

## Cohen's d = 0.62.

*Data are presented as means ± standard deviation for continuous variables, as number and percentage (%) for categorical variables*.

Comparing patients in the LOEU-CN (*n* = 19) and CN (*n* = 11) groups, no differences in age, gender, education, CSF biomarkers, and neuropsychological assessment were found (Table 3). As expected, EEG abnormalities were exclusively found in LOEU patients (26.3%, *p* < 0.05).

**Table 3:** Characteristics of patients with normal cognition depending on disease group.

		**LOEU-CN**	**CN**
*n*		19	11
Gender (female), *n* (%)		7 (36.8%)	4 (36.4%)
Age at seizure onset, mean ± SD (years)		65.1 ± 6.8	65.7 ± 7.7
Education, mean ± SD (years)		11.1 ± 4	9.8 ± 4
EEG abnormalities, *n* (%)		5 (26.3%)	0 (0%)^*^
CSF biomarkers, mean ± SD			
	Aβ42 (pg/mL)	1387.8 ± 671.6	1022.4 ± 196.3
	t-tau (pg/mL)	247.4 ± 92.1	298.5 ± 135
	p-tau (pg/mL)	43.1 ± 11.3	49.7 ± 20.1
	Aβ42/p-tau ratio	31 ± 8.7	24.9 ± 13.1
Neuropsychological assessment scores, mean ±SD			
	MMSE	28.3 ± 1.5	27.5 ± 1.6
	CDT	1.2 ± 1.6	1.1 ± 1.9
	DIGIT F	6.3 ± 1.3	6.1 ± 1.9
	DIGIT B	4.6 ± 0.9	4.7 ± 1.5
	RAVLT imm	39.7 ± 8	41.6 ± 8
	RAVLT del	7.9 ± 2.6	8.4 ± 2.3
	RAVLT FP	1.4 ± 2.1	1.1 ± 1.2
	TMT A	38.4 ± 11.5	42.8 ± 21
	TMT B	123 ± 46.4	131.3 ± 55.6
	TMT B-A	90.9 ± 32.4	90.8 ± 52.9
	FAB	16.6 ± 1.4	15.3 ± 3.6
	FAS	35.6 ± 9.4	41.4 ± 13.1
	CF	36.1 ± 7.6	41.3 ± 10.3
	PM'47	29.8 ± 3.6	30.8 ± 3.4
	CD	9.8 ± 1.7	9.7 ± 1.1
	CD L	67.8 ± 3.8	67 ± 2.1

LOEU, late onset epilepsy of unknown etiology; CN, cognitively normal; SD, standard deviation; EEG, electroencephalogram; CSF, cerebrospinal fluid; MMSE, Mini Mental State Examination; CDT, Clock Drawing Test; Digit F, Digit Span Forward; Digit B, Digit Span Backward; RAVLT, Rey Auditory Verbal Learning Test (Imm, Immediate Recall; Del, Delayed Recall; FP, False Positives). TMT, Trail Making Test; FAB, Frontal Assessment Battery; FAS, Phonemic/Letter Fluency (F,A,S); CF, Category Fluency; PM'47, Raven Colored Progressive Matrices'47; CD, Copying Drawings; CD L, Copying drawings with landmarks.

* p < 0.05.

Categorical variables were compared with χ^2^ test.

*Continuous variables were compared with t-test, with Bonferroni correction*.

Among people diagnosed with MCI, 27 were in the LOEU group (LOEU-MCI) and 21 in the NE-MCI group (Table 4). No significant differences in age, sex, and education were found between the two groups. Of note, epileptic abnormalities (sharp waves, spikes, or both, *n* = 15), and focal slowing (mainly frontal or temporal delta, *n* = 8) with standard visual assessment of EEG activity were exclusively found in the LOEU-MCI group (40.7%, *p* < 0.05).

**Table 4:** Characteristics of patients diagnosed with MCI at baseline (*n* = 48), depending on disease group.

		**LOEU-MCI**	**NE-MCI**
*n*		27	21
Gender (female), *n* (%)		13 (48.1%)	12 (57.1%)
Age at seizure onset, mean ± SD (years)		65.1 ± 6.8	65.7 ± 7.7
Education, mean ±SD (years)		8.3 ± 4.4	10.3 ± 4.4
EEG abnormalities, *n* (%)		11 (40.7%)	0 (0%)^*^
CSF biomarkers, mean ± SD			
	Aβ42 (pg/mL)	897.2 ± 347.9	820 ± 316.6
	t-tau (pg/mL)	355.1 ± 198.2	440.1 ± 238.7
	p-tau (pg/mL)	57.1 ± 29.1	70.4 ± 31.8
	Aβ42/p-tau ratio	21.9 ± 15.8	14.4 ± 8.8
AT(N) profile, *n* (%)	A+	9 (40.9%)	10 (47.6%)
	T+	6 (27.3%)	14 (66.7%)^*^
	A+/T-	4 (18.2%)	1 (4.8%)
	A+/T+	5 (22.7%)	9 (42.9%)
	A-/T+/N+	0 (0%)	3 (14.3%)
Neuropsychological assessment scores, mean ±SD			
	MMSE	25.6 ± 2.2^*^^#^	27.6 ± 1
	CDT	4.6 ± 3.1^*^^#^	0.9 ± 1.2
	DIGIT F	5.1 ± 1.1	5.9 ± 1.4
	DIGIT B	2.8 ± 1.5	3.7 ± 1.9
	RAVLT imm	28.8 ± 7.2	28.8 ± 8.5
	RAVLT del	4 ± 3.3	3.6 ± 3.2
	RAVLT TR	11.9 ± 2.7	11.8 ± 2.1
	RAVLT FP	4.7 ± 4.2	5.6 ± 4.6
	TMT A	83.8 ± 45	64.2 ± 30
	TMT B	218.8 ± 77.7	214 ± 90.9
	TMT B-A	115.3 ± 53.5	111.9 ± 66.1
	FAS	19.8 ± 7^*^^#^	34.3 ± 12.5
	CF	28.3 ± 7.8	29.1 ± 7.5
	PM'47	23.5 ± 4.6^*^^##^	27.2 ± 4.2
	CD	8.1 ± 2	9.2 ± 1.8
	CD L	58.2 ± 12.8	64.3 ± 4.8

LOEU, late onset epilepsy of unknown etiology; NE-MCI, non epileptic-mild cognitive impairment; SD, standard deviation; EEG, electroencephalogram; CSF, cerebrospinal fluid; MMSE, Mini Mental State Examination; CDT, Clock Drawing Test; Digit F, Digit Span Forward; Digit B, Digit Span Backward; RAVLT, Rey Auditory Verbal Learning Test (Imm, Immediate Recall; Del, Delayed Recall; TR, True Recognition; FP, False Positives). TMT, Trail Making Test; FAS, Phonemic/Letter Fluency (F,A,S); CF, Category Fluency; PM'47, Raven Colored Progressive Matrices'47; CD, Copying Drawings; CD L, Copying drawings with landmarks.

* p < 0.05,

^**^p < 0.01.

# Cohen's d > 1.0,

## Cohen's d = 0.77.

Categorical variables were compared with χ^2^ test.

*Continuous variables were compared with t-test, with Bonferroni correction*.

### Neuropsychological Findings

As expected, no differences were found between the LOEU-CN and CN groups on all neuropsychological scores. Furthermore, those scores were significantly worse in the LOEU-MCI than the LOEU-CN group in all domains explored (Table 2). Of note, the patients in the LOEU-MCI group performed significantly worse on MMSE, CDT, FAS and PM'47 compared with those in the NE-MCI group (*p* < 0.05, Table 4).

**Figure 1:**
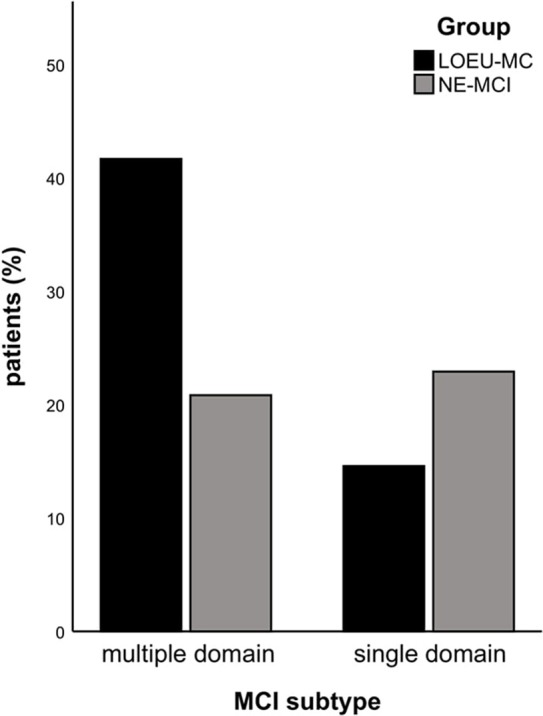
Distribution of mild cognitive impairment (MCI). LEOU, late onset epilepsy of unknown etiology; NE-MCI, non epileptic-mild cognitive impairment.

**Figure 2:**
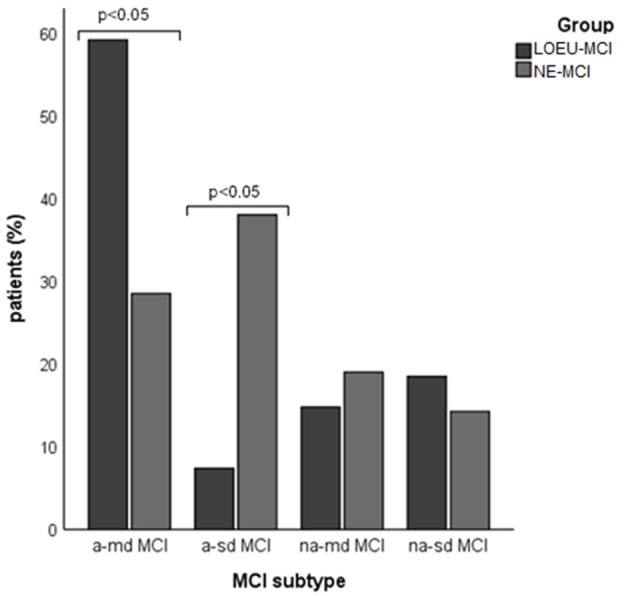
Distribution of mild cognitive impairment (MCI) subtype across groups. a-md MCI, amnestic multi-domain MCI; a-sd MCI, amnestic single-domain MCI; LEOU, late onset epilepsy of unknown etiology; NE-MCI, non epileptic-mild cognitive impairment; na-md MCI, non-amnestic multi-domain MCI; na-sd MCI, non-amnestic single-domain MCI.

Patients in the LOEU-MCI group exhibited a different distribution of MCI subtypes compared with those in the NE-MCI group (Figure 1). Specifically, people with LOEU-MCI were 3-fold more likely to suffer from multi-domain cognitive impairment compared with NE-MCI (OR 3.14, 95%CI 0.93–10.58, *p* = 0.06). On the contrary, single-domain MCI had marginally significant lower prevalence in the LOEU-MCI group than in the NE-MCI group (*n* = 7, 25.9% vs. *n* = 11, 52.4%, *p* = 0.07). Moreover, amnestic multi-domain MCI was significantly more frequent in the LOEU-MCI than in the NE-MCI group (*n* = 16, 59.3% vs. *n* = 6, 28.6%, *p* = 0.04) (Figure 2).

Comparing patients with multi-domain (*n* = 30) and single-domain (*n* = 18) MCI between the LOEU-MCI and NE-MCI groups, no differences in education and gender distribution were found (Table 5). Among patients with multi-domain MCI, those in the LOEU-MCI group had significantly worse performance on MMSE, CDT, and on FAS compared with those in the NE-MCI group (*p* < 0.05). Finally, among patients with single-domain MCI, those in the LOEU-MCI group showed worse performances in cognitive assessment, especially in FAS and CD (*p* < 0.05), compared with those in the NE-MCI group (Table 5).

**Table 5:** CSF and neuropsychological test scores in patients with baseline MCI in the LOEU-MCI and NE-MCI.

		**MCI**			
		**Multiple Domain**		**Single Domain**	
		**LOEU-MCI**	**NE-MCI**	**LOEU-MCI**	**NE-MCI**
*n*		20	10	7	11
Gender (female), *n* (%)		11 (55%)	7 (70%)	2 (28.6%)	5 (45.5%)
Age at seizure onset, mean ±SD (years)		71.2 ± 5.4	71.8 ± 5.9	64.7 ± 6.8	65.4 ± 7.6
Education, mean ±SD (years)		7.8 ± 4.4	7.5 ± 3.2	9.9 ± 4.6	12.9 ± 3.8
CSF biomarkers, mean ± SD					
	Aβ42	844.7 ± 341.8	780.1 ± 314.3	1,037.3 ± 354.0	856.4 ± 329.4
	t-tau	382.7 ± 223.9	433.2 ± 292.0	281.5 ± 74.3	446.5 ± 192.7
	p-tau	60.6 ± 33.3	68.2 ± 37.5	47.9 ± 9.2	72.4 ± 27.3
	Aβ42/p-tau ratio	21.5 ± 17.7	15.0 ± 10.2	23.0 ± 10.5	13.9 ± 7.7
AT(N) profile, *n* (%)					
	A+	8 (50.0%)	5 (50.0%)	1 (16.7%)	5 (45.5%)
	T+	6 (37.5%)	6 (60.0%)	0 (0%)^A^	8 (72.7%)
	A+/T-	3 (18.8%)	0 (0%)	1 (16.7%)	1 (9.1%)
	A+/T+	5 (31.3%)	5 (50.0%)	0 (0%)	4 (36.4%)
	A-/T+/N+	0 (0%)	1 (10%)	0 (0%)	2 (18.2%)
Neuropsychological assessment scores, mean ± SD					
	MMSE	25.2 ± 2.1^**A#**^	27.2 ± 0.6	26.7 ± 1.9	27.9 ± 1.1
	CDT	5.0 ± 3.1^A**#**^	1.3 ± 1.6	2.5 ± 3.5	0.6 ± 0.5
	DIGIT F	4.9 ± 1.0	5.5 ± 1.6	5.8 ± 1.3	6.3 ± 1.1
	DIGIT B	2.4 ± 1.4	2.9 ± 1.7	4.0 ±.8	4.6 ± 1.9
	RAVLT imm	26.7 ± 5.3	29.9 ± 10.4	34.4 ± 9.0	27.8 ± 6.8
	RAVLT del	3.3 ± 3.1	4.1 ± 3.7	5.9 ± 3.0	3.2 ± 2.8
	RAVLT TR	11.6 ± 2.8	13.0 ± 1.4	12.9 ± 2.2	11.4 ± 2.2
	RAVLT FP	5.2 ± 4.4	7.0 ± 4.2	3.4 ± 3.5	5.1 ± 4.9
	TMT A	92.5 ± 47.9	75.4 ± 32.0	57.5 ± 22.8	51.6 ± 23.3
	TMT B	238.1 ± 52.1	275.4 ± 49.1	154.3 ± 126.2	152.6 ± 81.7
	TMT B-A	133.3 ± 42.9	171.7 ± 63.8	43.5 ± 2.1	86.3 ± 51.4
	FAS	19.1 ± 6.7^**A##**^	28.9 ± 8.7	21.6 ± 8.1^**A**^	39.3 ± 13.6
	CF	27.0 ± 8.1	25.6 ± 6.6	31.6 ± 6.6	33.4 ± 6.5
	PM'47	22.2 ± 3.6	25.3 ± 3.8	26.3 ± 5.5	29.1 ± 3.8
	CD	7.9 ± 2.3	8.1 ± 1.6	8.6 ± 1.1^**a#**^	10.3 ± 1.2
	CD-L	55.6 ± 13.8	61.5 ± 4.2	65.8 ± 3.9	67.4 ± 3.3

LOEU, late onset epilepsy of unknown etiology; NE-MCI, non epileptic-mild cognitive impairment; SD, standard deviation; EEG, electroencephalogram; CSF, cerebrospinal fluid; MMSE, Mini Mental State Examination; CDT, Clock Drawing Test; Digit F, Digit Span Forward; Digit B, Digit Span Backward; RAVLT, Rey Auditory Verbal Learning Test (Imm, Immediate Recall; Del, Delayed Recall; TR, True Recognition; FP, False Positives). TMT, Trail Making Test; FAS, Phonemic/Letter Fluency (F,A,S); CF, Category Fluency; PM'47, Raven Colored Progressive Matrices'47; CD, Copying Drawings; CD L, Copying drawings with landmarks.

Results are based on two-sided tests. Pairs (LOEU vs. non-epileptic MCI) in each group (single or multiple domain) are compared. Tests are adjusted for all pairwise comparisons using the Bonferroni correction. a < 0.05, A < 0.01.

# Cohen's d > 1.0,

## *Cohen's d = 0.76*.

### CSF Biomarkers Findings

Among CSF biomarkers, mean Aβ42/p-tau ratio was consistently lower in the NE-MCI group compared to LOEU and CN patients (14.4 vs. 24.3 and 24.9 respectively, *p* < 0.05) (Table 1). Aβ42 was significantly lower in LOEU-MCI compared to LOEU-CN (897.2 pg/ml vs. 1387.8 pg/ml, *p* < 0.05). In particular, all patients in the LOEU-CN group showed normal Aβ42 values, while nine (41%) in the LOEU-MCI group showed Aβ42 decrease (*p* < 0.05) (Table 2).

Comparing LOEU-MCI vs. the NE-MCI, despite no differences in mean CSF biomarkers levels (*p* > 0.05), amyloid pathology (A+) was similar across groups, while tauopathy was strictly predominant in the latter (66.7% vs. 27.3%, *p* < 0.01) (Table 4). An AD-like CSF profile (A+/T+) was found in 42.9% of NE-MCI patients vs. 22.7% of LOEU-MCI patients (*p* = 0.16), and an A-/T+/N+ status was almost significantly restricted to NE-MCI patients (14.3% vs. 0% in LOEU-MCI, *p* = 0.06) (Table 4). Comparing LOEU-MCI vs. NE-MCI among multi-domain and single-domain MCI, no significant differences were found in mean CSF biomarkers. However, a higher occurrence of tauopathy among single-domain non-epileptic MCI emerged compared to LOEU-MCI (0% vs. 72.7%, *p* < 0.01) (Table 5).

### EEG Source Estimates

**Figure 3:**
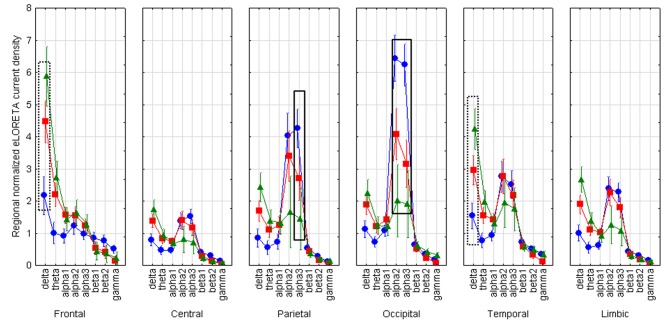
Statistical ANOVA for interaction among group, band and EEG region of interest. LOEU, late onset epilepsy of unknown etiology; LOEU-MCI, patients with late onset epilepsy and mild cognitive impairment; NE-MCI, non epileptic-mild cognitive impairment. Official exact low-resolution brain electromagnetic tomography (eLORETA) freeware was used to estimate resting-state (rs)-EEG normalized cortical sources delta, theta, alpha 1, alpha 2, alpha 3, beta 1, beta 2, gamma. Mean values (± standard error, SE) of eLORETA cortical source activity of resting state eyes-closed EEG rhythms for (i) Group (CN, NE-MCI, LOEU-MCI), (ii) Band (delta, theta, alpha1, alpha2, alpha3, beta1, beta2 and gamma), and (iii) ROI (central frontal, parietal, occipital, temporal, limbic). Compared to the non-epileptic MCI group, LOEU with MCI group is characterized by an amplitude increase of frontal and temporal delta sources (*p* < 0.05) and an amplitude decrease of occipital alpha 2 sources, parietal and occipital alpha 1 sources (*p* < 0.05).

LOEU-MCI, NE-MCI, and CN groups showed similar age, gender, education, and CSF biomarkers. Estimates of rsEEG sources revealed significant differences in frequency and topographic features among those groups (ANOVA *p* < 0.001). Compared to CN and NE-MCI, LOEU-MCI exhibited a significant increase in activation in frontal and temporal delta sources (*p* < 0.05). Moreover, the LOEU-MCI group also showed a significant decrease in the activation in occipital alpha 2 as well as parietal and occipital alpha 1 sources compared with both CN and NE-MCI groups (*p* < 0.05). NE-MCI had increased delta sources in frontal and temporal regions compared to CN (*p* < 0.05), and higher alpha 2 in occipital regions (Figure 3). These results were confirmed by the lack of outliers as revealed by the Grubb's test.

## Discussion

Despite the higher risk of developing cognitive impairment and dementia in subjects with epilepsy, we still lack data on cognitive performance and tools to stratify the risk of decline in LOEU (4, 14, 15, 17, 35). In this observational, cross-sectional comparative study, we delineated cognitive performance, CSF AD biomarkers profile, and resting-state EEG cortical rhythms in patients with LOEU, comparing them to non-epileptic controls, including NE-MCI and CN subjects. Our results highlight that MCI status is relatively frequent in LOEU patients, namely 59% of cases in our consecutive series. Compared with LOEU patients without cognitive deficits, those with LOEU-MCI suffers from amyloidosis as revealed by the β-amyloid decrease in the CSF. Compared with the MCI patients without epilepsy, LOEU-MCI shows prominent abnormalities in multiple cognitive domains as well as delta and alpha sources of EEG rhythms related to quiet vigilance. Therefore, a role for β-amyloid can be hypothesized, driving both epileptogenesis and cognitive decline (14, 15, 17, 36).

In this study, MCI emerged in 59% of LOEU patients at the time of epilepsy diagnosis. Our finding is in line with a previously reported observational study, which, using Epitrack, detected cognitive impairment in up to 58% of LOE (16). However, that study lacked a control group, and only used Epitrack to assess cognitive function, with consequent limitations on domain-specific ascertainment. Therefore, our study, with strict enrollment criteria, multiple comparisons with control groups, and standardized and comprehensive neuropsychological assessment, adds to previous literature, suggesting that cognitive impairment already happens at epilepsy diagnosis, with deficits not restricted to executive functions. Our finding, emerging from consecutive enrollment of thoroughly characterized LOEU patients, confirms a relatively high prevalence of cognitive impairment in adult patients with epilepsy, who are therefore to be considered as a population at very high risk of cognitive decline, and so these patients need to be thoroughly screened (10–13). Moreover, LOEU-MCI patients seem to have a peculiar pattern of cognitive impairment, with multi-domain MCI being three times more frequent compared to NE-MCI. Indeed, LOEU-MCI is associated with worse cognitive performance on measures of global cognition, visuospatial abilities, and executive functions compared to NE-MCI. These findings, that have emerged despite the small sample size, are strengthened by the marginal role attributable to the antiepileptic treatment initiated, and point to plausible direct influence of epilepsy on cognitive functioning. To the latter extent, our results also suggest that an underlying process might drive both epileptogenesis and cognitive impairment (14). Indeed, CSF biomarkers profiling highlights an increased prevalence of β-amyloid pathology among patients with LOEU-MCI compared to LOEU-CN. Such data, together with the similar prevalence of tau pathology, suggests that β-amyloid might represent a common ground on which epileptogenesis, and cognitive decline develop, plausibly, hand in hand. Such hypothesis is also supported by comparing the A/T/(N) CSF profile between LOEU-MCI and NE-MCI. Indeed, while amyloid pathology and the AD-like CSF profile (A+/T+) were similar across groups, non-AD pathologic changes were infrequent in LOEU-MCI, denoting a possible divergence between the mechanisms leading to MCI across groups; on the epileptic side, amyloid pathology might drive epileptogenesis and cognitive impairment, while on the other side, other non-amyloid related processes may contribute to MCI status. Such findings are in line with our previous reports of an increased burden of β-amyloid pathology in patients with LOEU (14, 15, 17), and call for a need of further collaborative studies, with large samples and a standardized CSF biomarker assessment (including Aβ42/Aβ40 ratio) to explore the intertwining of LOEU and dementia.

Finally, the findings of the present rsEEG study opened a window on the neurophysiological underpinning of the regulation of quiet vigilance in LOEU-MCI patients. Here we report that parietal and occipital sources of alpha (8–12 Hz) rhythms were less active in the LOEU-MCI than the NE-MCI and CN groups and the opposite was true for frontal and temporal cortical delta sources. Abnormality in the alpha source connectivity has been documented in AD, even at the stage of MCI, with decreasing posterior alpha peak amplitude associated with worsening cognitive functioning at follow-up (37). Therefore, results from rsEEG in our study suggest that LOEU-MCI already present a surrogate marker for worsening cognitive function, possibly reflecting cholinergic impairment in prodromal state of cognitive decline (38). Indeed, it can be speculated that these findings might echo the effects of Alzheimer's neuropathology on the synchronization of cortical neurons targeted by thalamocortical and basal forebrain-hippocampus-cortical circuits, underpinning the neurophysiological control of human brain arousal (39, 40). Since delta and alpha sources of rsEEG rhythms were found to be abnormal in AD patients in relation to CSF biomarkers and structural abnormalities (33, 38, 41–44), rsEEG might represent a tool for the early stratification of the risk of cognitive decline among epileptic patients, to be tested in future studies.

### Limitations and Strengths of the Study

First, despite the fact that consecutive enrollment was pursued, selection bias could have occurred, since all enrolling centers are tertiary centers for referral. However, given the consistent sample of LOEU patients reported, our cohort is indeed likely to grossly represent the general population suffering from LOEU. At the same time, the limited sample allowed us to provide extensive and standardized neuropsychological screening, in a no-funding environment. Second, our study lacks longitudinal follow-up. However, the aim of this study was clearly cross-sectional, with profiling of LOEU patients at diagnosis. Longitudinal prospective studies are needed to finally define the strength of our preliminary findings. A further limitation of the study is the possible adverse effects of antiseizure medications on cognitive function (36). However, no major concern directly related to antiseizure medications arose, and all testing happened before/at antiseizure medication initiation, further supporting the reliability of our results.

In summary, our study highlights that MCI status is relatively frequent in LOEU patients, involves multiple cognitive domains, and might be driven, at least in part, by amyloid pathology. LOEU-MCI status is associated with abnormalities in cortical sources of EEG rhythms known to correlate with cognitive worsening and might therefore represent a useful tool to consider to predict the risk of dementia, together with CSF biomarkers profile. Future prospective, longitudinal, and multicenter studies in a larger cohort of consecutive LEOU patients with and without MCI status will have to cross-validate these findings and test the value of the above CSF and EEG variables in the prediction of their cognitive decline and functional capacity over time.

## Data Availability Statement

The datasets generated for this study are available on request to the corresponding author.

## Ethics Statement

All patients gave written informed consent and that ethical approval was granted by our Ethical Board (WP5 P001; N 2049/12).

## Disclosure

LP served as Member of Advisory Boards for Fujirebio, IBL, Roche and Merck. The other authors have no conflict to declare.

## Author Contributions

EN and NS conceived the study idea and collected data. LF supervised drafting, data acquisition, and revised the manuscript. MR participated in data cleaning, statistical analysis, interpretation, and drafting of results. JD, FD, VI, and LT collected data. GN, RL, MP, and CD performed the EEG data analysis for the eLORETA source estimation, the statistical comparisons of the EEG source estimates, and the preparation of the related iconography and main text. CC and CB contributed to the study design and the production of the main text of the manuscript. JD and LP revised the manuscript.

### Conflict of Interest

The authors declare that the research was conducted in the absence of any commercial or financial relationships that could be construed as a potential conflict of interest.
